# Accessory Cavitated Uterine Malformation (ACUM): An Incidental Finding in Early Pregnancy

**DOI:** 10.7759/cureus.98510

**Published:** 2025-12-05

**Authors:** Catarina C Neves, Rodrigo P Mata, Ana Codorniz, Fernando Guerreiro

**Affiliations:** 1 Obstetrics and Gynecology Department, Unidade Local de Saúde do Algarve - Portimão Unit, Portimão, PRT

**Keywords:** accessory cavitated uterine malformation, congenital uterine malformation, dysmenorrhoea, early pregnancy, first trimester ultrasound, gynaecological ultrasound

## Abstract

Accessory cavitated uterine malformation (ACUM) is a relatively rare and underdiagnosed Müllerian anomaly. We report an ACUM identified incidentally at first-trimester screening in a woman in her early 30s with a spontaneously conceived pregnancy and a history of mild dysmenorrhoea. Transabdominal ultrasound demonstrated a well-circumscribed intramyometrial cavitated lesion beneath the left round ligament, initially anechoic and later with a small echogenic component, without communication with the endometrial cavity and with normal ovaries. The pregnancy progressed uneventfully and culminated in an uncomplicated term vaginal delivery. Postpartum imaging showed persistence of the lesion without change, and the patient remains asymptomatic under gynaecological surveillance. This case highlights the diagnostic challenge of ACUM in pregnancy and emphasises the need for uniform diagnostic criteria as well as prospective studies to define its natural history, reproductive impact, and optimal management.

## Introduction

Accessory cavitated uterine malformation (ACUM) is a rare Müllerian anomaly characterised by an isolated, cavitated myometrial lesion, most often near the insertion of the round ligament in women with normal uterine anatomy [1]. Its incidence and prevalence are unknown, and the condition is likely still underdiagnosed [2], as it has only recently been recognised as a distinct clinical entity. Multiple definitions have been proposed over the years to describe this lesion, and various terms have been used in the literature, including adenomyotic cyst, juvenile cystic adenomyosis, myometrial cyst and uterine-like mass [3]. Despite this, ACUM remains unclassified in the current uterine anomaly classifications proposed by the European Society of Human Reproduction and Embryology (ESHRE), the European Society for Gynaecological Endoscopy (ESGE) [4] and the revised American Society for Reproductive Medicine (ASRM) [5].

We report a rare case of an ACUM incidentally identified in early pregnancy, an unusual finding that underscores the diagnostic and imaging challenges of this entity during gestation.

## Case presentation

A woman in her early 30s, gravida 2 para 1, with a history of an uncomplicated spontaneous vaginal delivery approximately three years earlier, attended a routine first-trimester ultrasound for a spontaneously conceived pregnancy. She reported regular menstrual cycles every 28 days, lasting five days, with normal flow but with mild dysmenorrhoea. She had used a combined oral contraceptive pill for more than 10 years in total to control dysmenorrhoea. The patient had no significant past medical or gynaecological history and was a current smoker, consuming around 10 cigarettes per day.

**Figure 1:**
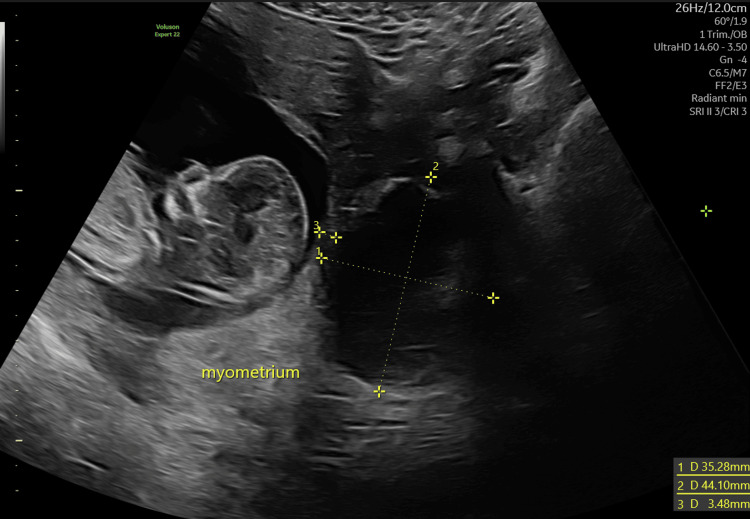
Transabdominal ultrasound at 13 weeks + two days (first trimester), transverse uterine view showing a viable intrauterine pregnancy and a predominantly anechoic mass with two orthogonal measurements and its distance from the amniotic cavity.

First-trimester ultrasound at 13 weeks and two days of gestation demonstrated a single intrauterine pregnancy with the crown-rump length consistent with gestational age. Combined screening for aneuploidy and early-onset pre-eclampsia indicated a low-risk result. During the examination, an incidental anechoic cystic mass measuring 3.5 × 4.4 × 3.7 cm, with well-defined margins and a colour Doppler score of 1, was noted in the left uterine wall, without a clear cleavage plane from the myometrium and located 3.5 mm from the amniotic cavity (Figure 1). Furthermore, both ovaries were clearly identified, with normal size and follicular pattern and no pathological findings.

**Figure 2:**
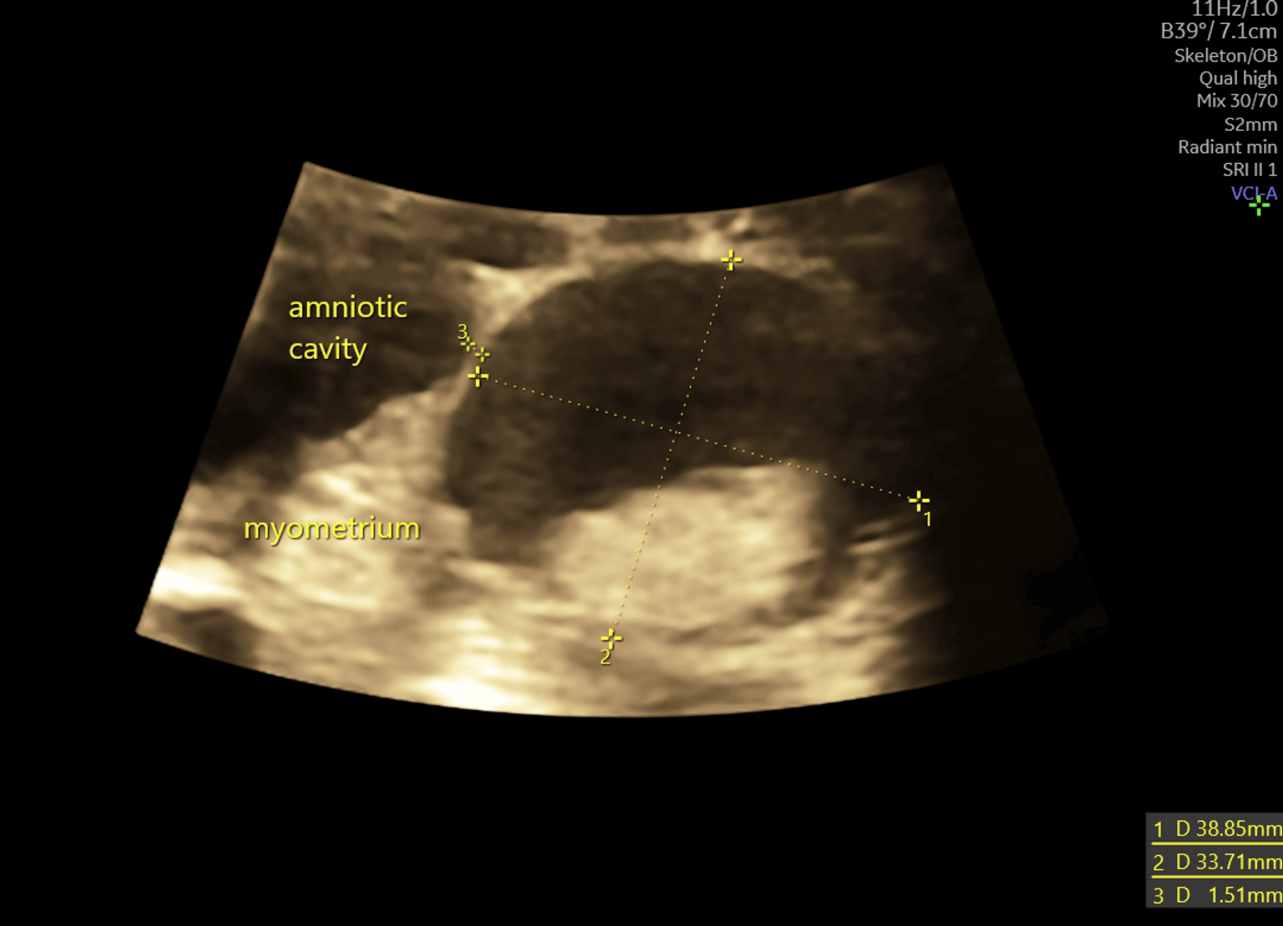
Transabdominal ultrasound at 22 weeks + five days (second trimester), transverse uterine view demonstrating an ACUM with hyperechoic content, measurements in two orthogonal planes, and the distance from the amniotic cavity. ACUM: accessory cavitated uterine malformation

Second-trimester ultrasound at 22 weeks and five days confirmed a viable fetus, and no structural fetal abnormalities were detected. The previously described cystic lesion persisted, measuring 3.9 × 3.3 × 3.5 cm and located 1.5 mm from the amniotic cavity, with well-defined margins and similar overall features, except for the appearance of an echogenic internal component without vascularisation (Figure 2).

The pregnancy had been closely monitored, without complications. She delivered spontaneously at term a healthy male newborn weighing 3180 g, with Apgar scores of 9, 10, and 10 at one, five, and 10 minutes, respectively. Estimated blood loss was within normal limits, and the perineum remained intact. The postpartum course was uncomplicated, and the patient was discharged on the second day after delivery.

A follow-up ultrasound performed two months postpartum demonstrated persistence of the lesion with unchanged characteristics. The patient remains asymptomatic and under regular gynaecological surveillance.

## Discussion

Over the years, several authors have proposed slightly different diagnostic criteria for ACUM. According to Acién et al. [6], in 2010, ACUM is an isolated accessory cavitated mass, with a normal uterus, tubes and ovaries. Histopathology demonstrates an accessory intramyometrial cavity lined by functional endometrium (epithelium, glands and stroma) containing thick altered haemorrhagic material, with no diffuse adjacent adenomyosis (aside from occasional small foci).

Naftalin et al. [1], in 2021, subsequently proposed sonographic criteria, describing ACUM as a cavitated lesion with a myometrial mantle and echogenic contents, located in the anterolateral wall of the myometrium under the insertion of the round ligament. It’s crucial for the diagnosis of the exclusion of other obstructive Müllerian anomalies, such as communicating or non-communicating uterine horns. Despite increasing recognition, relatively few publications have addressed this entity; a recent review published in 2024 [3] identified 53 articles comprising 115 cases that met the minimal criteria for ACUM described in the literature.

Although diagnostic uncertainty remained, the present case fulfils the sonographic appearance previously described, corresponding to a cavitated mass within the myometrium, with echogenic content and located in the left anterolateral uterine wall. There was no evidence of other associated uterine malformations, no communication with the endometrial cavity and the ovaries appeared normal.

Although its precise aetiology is still uncertain, ACUM is considered a congenital malformation resulting from ectopia or duplication and persistence of the Müllerian ductal tissue adjacent to the round-ligament insertion, potentially linked to dysfunction of the female gubernaculum [6].

Severe dysmenorrhoea and chronic pelvic pain are the most frequent but nonspecific symptoms of ACUM, often leading to under-recognition and delayed diagnosis [7]. These symptoms are typically felt at the side of the lesion and result from cyclic bleeding within the accessory cavity, causing progressive distension and intracavitary pressure [8]. In our case, the patient reported mild dysmenorrhoea, managed for several years with a combined oral contraceptive pill, without any prior diagnosis of a uterine anomaly.

The diagnosis of ACUM is based on imaging techniques, with transvaginal or transabdominal ultrasound representing the primary diagnostic tool. Three-dimensional ultrasound can improve visualisation of uterine morphology and help exclude other uterine malformations [3]. Magnetic resonance imaging (MRI) may provide additional anatomical detail, but is generally reserved for equivocal cases, while hysterosalpingography has a limited role and is mainly used to rule out obstructive anomalies [1].

Diagnosis of ACUM during pregnancy is particularly challenging because imaging findings may overlap with other uterine pathologies such as non-communicating functional rudimentary uterine horn, juvenile adenomyosis or adenomyotic cyst, intramural uterine leiomyoma with cystic degeneration, and intramyometrial or subserosal endometrioma [3]. In pregnancy, ACUM is usually detected incidentally or in the context of pain, and differentiation from these conditions is essential. Histopathological confirmation remains the diagnostic gold standard but is rarely performed during pregnancy. Reports of ACUM diagnosed in pregnancy are exceedingly rare. Naftalin et al. [1] identified three cases among 20 women with ACUM; all conceived spontaneously, two had uneventful term vaginal deliveries, and one experienced a first-trimester miscarriage. The present case adds to the limited descriptions of ACUM identified during pregnancy, emphasising the diagnostic difficulty of this entity in the gestational setting.

The treatment of ACUM depends on symptom severity and reproductive goals. Surgical excision, often performed laparoscopically, is the standard approach for symptomatic cases and typically results in complete resolution of pain and dysmenorrhoea. Uterine-sparing techniques are preferred in women wishing to preserve fertility [1,3]. In rare cases, alternative approaches such as ultrasound-guided alcohol sclerotherapy have been reported [9]. Conservative management is appropriate for asymptomatic women or for those diagnosed incidentally during or after pregnancy, as in the present case. Limited data suggest that expectant management is generally safe, although the long-term reproductive and obstetric outcomes of untreated ACUM remain poorly defined [10]. Further studies are needed to clarify the natural history of the condition and to establish optimal management strategies.

## Conclusions

In conclusion, ACUM is a relatively rare and likely underdiagnosed malformation with limited literature available and few reports during pregnancy. This case highlights the diagnostic uncertainty and imaging challenges of identifying ACUM in the gravid uterus. Greater recognition of this condition could improve diagnosis and care for women with chronic pelvic pain and dysmenorrhoea. Further case reporting and focused studies are needed to define its natural history, reproductive impact, and optimal management.
